# Local stakeholders’ perceptions of community sensitization for school-based deworming programme in Kenya

**DOI:** 10.1186/s40794-017-0058-9

**Published:** 2017-08-22

**Authors:** D. W. Njomo, J. Masaku, F. Mwende, G. Odhiambo, R. Musuva, E. Matey, I. G. Thuita, J. H. Kihara

**Affiliations:** 1 Eastern & Southern Africa Centre of International Parasite Control, Kenya Medical Research Institute, P.O. Box 54840-00200, Nairobi, Kenya; 2 Centre for Global Health Research, Kenya Medical Research Institute, P.O. Box 1578-40100, Kisumu, Kenya; 3 Centre for Microbiology Research, Kenya Medical Research Institute, P.O. Box 54840-00200, Nairobi, Kenya; 4 Ministry of Education, Science and Technology, Kenya, P.O. Box 30040-00100, Nairobi, Kenya; 5 Ministry of Health, Division of Vector Borne Diseases, P.O. Box 30016–00100, Nairobi, Kenya

**Keywords:** Community sensitization, Deworming, Soil-transmitted helminthes, Local, Stakeholders

## Abstract

**Background:**

In Kenya, the National School-Based Deworming Programme (NSBDP) for soil-transmitted helminthes and schistosomiasis in prioritized areas has been going on since the year 2012. By the year 2013 over 6 million School Age Children (SAC) had been treated. A community sensitization supplement containing key messages and answers to frequently asked questions was developed as a guiding tool. Awareness creation methods used include county sensitization meetings, stakeholder forums, town criers and posters. To assess the local stakeholders’ perceptions of community sensitization for programme implementation, a qualitative cross-sectional survey was conducted in four-sub-counties of coastal region.

**Methods:**

In-depth interviews (IDIs) were administered to 40 purposively selected opinion leaders so as to explore their perceptions of awareness creation sources, adequacy of information given, length of period of awareness creation and period between which information is given and drugs are administered. Separate IDIs were administered to pre-school teachers (41), community health extension workers (34) and primary school teachers (38). To elicit more information, 20 focus group discussions (FGDs) categorized by gender and age were conducted among parents of school-age children. Data was audio recorded, transcribed, coded and analyzed manually by study themes.

**Results:**

The most commonly reported source of information was school pupils. Due to low literacy levels, use of posters was regarded as ineffective and religious institutions, town criers and vernacular radio stations considered more effective. The information given during programme implementation was considered inadequate and use of complementary methods to reach all targeted children including the non-enrolled, and relay adequate information reported as important. Use of school and chief’s meetings with health personnel being present was mentioned as a useful method that would allow for interaction with participants indicating that they did not understand why adults were not being treated. Repeated awareness creation before deworming day to serve as a reminder and to reach those missing initial messages was also mentioned as important. Furthermore, the awareness creation period needed to be extended as 85% of the participants indicated that they learnt of deworming a day before it took place or after their children had received the drugs.

**Conclusion:**

Awareness creation is a key factor in the success of NSBDP implementation. For programme sustainability, preferences of local stakeholders need to be considered as control of worms can only be achieved through an integrated approach of deworming, health education and use of safe water and sanitation facilities which require collaboration with local stakeholders.

## Background

Neglected tropical diseases (NTDs) are a group of tropical diseases which cause disability, disfigurement, under nutrition and cognitive impairment and affect more than one billion poor people worldwide living at the periphery of health systems [1]. Kenya like many other African Countries [2] is implementing a national treatment programme for control of soil-transmitted helminthes (STHs), spp. *Ascaris lumbricoides*, *Trichuris trichiura* and hookworm which contribute the greatest disease burden among the NTDs [3]. Hookworm in Kenya occurs throughout most parts of the country, with highest prevalence being found in south-western Kenya and in Kilifi and Kwale counties on the Coast Region while *Ascaris lumbricoides* and *Trichuris trichiura* are most prevalent in western Kenya and in Lamu and Tana River also on the Coast [4].

The Kenya NSBDP was launched in 2009 with albendazole for STH treatment and in 2012 re-launched for both STH treatment and schistosomiasis with praziquantel administered in prioritized areas. Results of a baseline survey conducted in 2012 showed that overall, 32.4% of children were infected with at least one STH species, with *Ascaris lumbricoides* being the most common species detected [5]. Annual mass drug administration (MDA) using trained primary school teachers has been going on targeting all pre-school and school age children as recommended by World Health Organization [6] and pilot-tested in Kenya [7]. In the years 2012 and 2013, totals of 5,056,530 and 5,066,396 respectively of school-age children were dewormed with average treatment coverage of 78.6% and 76% for the respective 2 years being achieved [8]. Community sensitization to raise awareness about the programme and the importance of deworming, to maximize deworming uptake among the targeted population and to manage risks in case of adverse events or negative publicity is cascaded from National to school level. The strategies used to create awareness include the Community Health Extension Workers (CHEWs) who are charged with sensitizing the community members about cause, prevention and treatment of worm infestation, conduct mobilization before deworming day to maximize uptake. Other strategies used are school teachers who are meant to sensitize school enrolled and non-enrolled children as well as parents on the benefits of deworming, radio which is managed at county level and posters which are distributed at county meetings and teacher training sessions for visible mounting in public places. Regional Stakeholder Forums are also used with an aim of engaging external institutions not directly involved in the programme but which can play a key role in helping sensitize and mobilize children as well as in building a network of partners to maximize opportunities for collaboration and support.

The results presented in this paper are part of a larger study entitled “Evaluating Different Drug Delivery Approaches for Treatment of STHs among Pre-School Age Children during the National School-Based Deworming Programme of Kenya” which was conducted after annual treatment of the year 2014. The current study sought to assess the local stakeholders’ perceptions of the awareness creation activities conducted during NSBDP with a view of coming up with ways of improving the community sensitization and participation during programme implementation.

## Methods

### Study site

The study was conducted in four STHs endemic sub-counties (Matuga, Msambweni, Lunga Lunga and Malindi) in coastal Kenya [5]. Three of the sub-counties are located in Kwale County while Malindi sub-county is located in Kilifi County. Kwale County is located, 40 km south of Mombasa, the second largest city in Kenya, and has an area of 8360 km^2^ with a population of 649,931 persons [9] and lies at an altitude of between 60 and 135 m above sea level. The County has 3 hospitals, 5 health centers, 37 government dispensaries, and 3 private dispensaries. Accessibility of health services is however low. A majority of the population live over 5 km away from the nearest health facility. Shortage of drugs and lack of diagnostic facilities adversely affect provision of quality health care. Cost of health care system is also a barrier to access to services. The doctor/patient ratio stands at 1:82,690 which in itself is telling of services offered due to shortage of staff in the health facilities. The most prevalent diseases are; malaria, respiratory diseases, diarrhea, intestinal worms, stomachache and flu. The utilization of health facility for child delivery stands at 32% [10], the main reasons for low levels of use being distances to the nearest health facilities and low socio-economic status.

Malindi sub-county in Kilifi County is located 120 km northeast of Mombasa, and lies between latitudes 2.2^0^ and 4^0^ south and between longitudes 39^0^ and 41^0^ east. It covers a geographical area of 7605 km^2^ with a total population of 384,643 [9]. The sub-county has 3 hospitals (1government and 2 private); 24 dispensaries (17 governments and 7 Non-Governmental Organizations) and 4 private chemists. The average distance to the nearest health facility for urban areas is 1 km and 3 km for rural areas. Most of the health facilities are therefore not accessible to the majority of the population. High poverty levels, cost sharing and long distances inhibit people from visiting these facilities. The doctor/patient ratio is 1:19,502. The most prevalent diseases are; malaria, respiratory diseases, diarrhea, intestinal worms, sexually transmitted infections, anemia and eye infections. The utilization of health facility for child delivery is at 41% and reasons for low usage are distance and low socio-economic status [11].

### Study design and setting

This was a retrospective cross-sectional study that utilized qualitative methods for data collection. The data was collected in May 2014, after the February 2014 round of deworming.

### Study population

The study participants included: community health extension workers (34), pre-school teachers (41), primary school teachers (38) and opinion leaders; social, community and religious groups leaders (40) serving the four sub-counties who were purposively selected for the study. Separate in-depth interviews were administered to each category of informants so as to elicit information on their perceptions of community sensitization for the NSBDP. Additionally, 20 FGDs were conducted with community groups who are parents of school-age children characterized by age and gender. All the data were collected by trained KEMRI personnel. Notes were taken during the IDIs and FGDs and audiocassettes used to tape record all the information using *Kiswahil*i, a commonly used language in the study area. The interviewing and FGD process adhered to the principles of collecting qualitative data, which include forming a relaxed and trusting relationship with informants and FGD participants, encouraging participation, observing and noting non-verbal cues, silence and probing. The number of interviews and group discussion was determined by the level of saturation i.e. once no new information was being received, then no more interviews or group discussions were conducted [12, 13]. The tapes were later transcribed and translated into English.

### Data management and analysis

The qualitative data from various sources were analyzed manually according to the themes of the study which included perceptions of awareness creation sources, adequacy of information given, length of period of awareness creation and period between which information is given and drugs are administered. The data were then triangulated for cross-verification to help increase the credibility and validity of the results by continuously cross-checking the data from the various sources. Quantitative data from the socio-demographic profiles were managed using excel spreadsheets.

## Results

### Background characteristics of the study participants

A total of 34 CHEWs, 41pre-school teachers, 38 primary school teachers, and 40 opinion leaders participated in the separate in-depth interviews and a total of 20 FGDs were conducted amongst the community groups who are parents of school-age children stratified by age and sex. Table 1 presents the socio-demographic characteristics of the IDIs study participants while Table 2 of the FGDs study participants. The socio-demographic characteristics demonstrate the appropriateness of each category of participants for the study.

**Table 1:** Socio-demographic characteristics of the study participants in the IDIs

Description	Frequency (*N* = 154)	Percentage (%)
Gender		
Male	95	61.7
Female	59	38.3
Age in years		
20–24	8	5.2
25–29	21	13.6
30–34	25	16.2
35–39	15	9.7
40–44	20	12.9
45–49	24	15.6
≥ 50	41	26.6
Marital status		
Single	26	16.9
Married	126	81.8
Divorced	2	1.3
Religion		
Christianity	79	51.3
Islam	72	46.8
Missing	3	1.9
Occupation		
Chief/Assistant Chief	14	9.1
Business	5	3.2
Farmer	2	1.3
CHEW/PHO	34	22.1
Religious leader (Pastor or Imam)	5	3.2
School chairman	3	1.9
Village elder	9	5.8
Primary School Teacher	38	24.8
Pre-School Teacher	41	26.6
Youth leader	2	1.3

**Table 2:** Socio-demographic characteristics of the study participants in the FGDs

Description	Frequency (*N* = 203)	Percentage (%)
Gender		
Male	89	43.8
Female	100	49.3
Missing	14	6.9
Age in years		
18–19	2	1.0
20–24	36	17.7
25–29	30	14.8
30–34	39	19.2
35–39	35	17.2
40–44	18	8.9
45–49	18	8.9
≥ 50	24	11.8
Missing	1	0.5
Educational level		
Primary education^a^	105	51.7
Secondary education^a^	14	6.9
None	47	23.2
Missing	37	18.2
Religion		
Christianity	89	43.8
Islam	107	52.7
None	4	2.0
Missing	3	1.5
Occupation		
Business	40	19.7
Farming	111	54.7
Fishing/Fish monger	3	1.5
Housewife	27	13.3
Casual laborer	9	4.4
Religious leader (Pastor or Imam)	3	1.5
Community health volunteer	1	0.5
Skilled laborer	2	1.0
Village elder	1	0.5
Teacher	3	1.5
Missing	3	1.5

^a^Includes people who received some education but may not have completed this level

In the following section perceptions of the study participants are presented under the following four headings: sources of information on school-based deworming; perception of adequacy of information given; perception of length of awareness creation on school-based deworming and perception on length of period between which information is given and drugs are administered.

### Source of information on school-based deworming

The most common source of information among all groups of study participants was the school pupils. All 38 primary school teachers indicated that the community had been made aware of the deworming activity with close to three-fifths(*n* = 28) indicating that they made announcements at school and instructed the pupils to take the message home with only five (5) indicating that they held parents’ meetings in the school. Less than one-third (*n* = 12) of the pre-school teachers reported that they were informed about the programme by the primary school teachers.

About a half (*n* = 19) of the opinion leaders and of the CHEWs (*n* = 18) indicated that the community got the information from school pupils. Furthermore 75% of the participants in 12 FGDs indicated that they were informed about the activity by the school children. However, in 8 FGDs, more than two-thirds of the participants indicated that they were not aware and only knew after their children had been dewormed.

A 40 year old male opinion leader in Malindi sub-county thus stated: “*The head teachers sent the children with information on the deworming but not every person in the community has children in school.”*

In Malindi sub-county, a 54 year old male CHEW further stated that*: “Teachers told the children to tell their parents that they were going to get deworming tablets in school.”*

In Matuga sub-county, a 33 year old female primary school teacher stated that: “*After the training we create awareness to the pupils, who inform their parents when they go home, and also inform other community members who may have non-enrolled pupils.”*

Other sources of information mentioned included community leaders (Table 3) and health personnel (Table 4).

**Table 3:** Community leaders as source of information on school-based deworming

Study Participants	Frequency of Mention
Opinion Leaders (IDIs)	14
CHEWs (IDIs)	15
Primary School Teachers (IDIs)	7
Pre-School Teachers (IDIs)	4
Parents (FGDs)	6

**Table 4:** Health personnel as source of information on school-based deworming

Study Participants	Frequency of Mention
Opinion Leaders (IDIs)	6
CHEWs (IDIs)	8
Primary School Teachers (IDIs)	2
Pre-School Teachers (IDIs)	10
Parents (FGDs)	4

Less than one-third (*n* = 11) of the teachers furthermore mentioned that they used posters which they had received during the trainings and which they mounted at trading centers. It was only in 2 FGDs where participants indicated that they learned about the programme through posters. Due to low literacy levels, use of posters was regarded as ineffective and four-fifths of the participants felt that use of religious institutions, town criers and vernacular radio stations would be more effective.

A 32 year old female primary teacher in Kwale sub-county stated: “*We sent the pupils, as most of the parents are illiterate, posters are not good, most parents are ignorant.*”

About one-sixth (*n* = 6) of the CHEWs and about one-fifth (*n* = 6) of the opinion leaders however indicated that they could not comment on the source of information as they were not involved and were not aware of how sensitization activities were conducted.

### Perceptions on adequacy of information given

Eighty percent of participants in all study groups indicated that the information given was inadequate and expressed the importance of using complementary methods as not all targeted children are enrolled in school and school pupils are likely to forget or relay incorrect messages.

A half (*n* = 20) of the opinion leaders were of the perception that the information given was inadequate as they did not understand about the programme and there were no reminders made with one eighth (*n* = 5) indicating that they could not comment about information adequacy as they were not involved in community sensitization. Only about three-eighths (*n* = 14) of the opinion leaders however thought that the information given during community sensitization was adequate.

Slightly more than a half (*n* = 19) of the CHEWs indicated that the information given was inadequate, while only about one-quarter (*n* = 9) stated that the methods were adequate with six (6) indicating that they could not comment as they were not involved.

A majority (*n* = 30) of the primary school teachers indicated that there is need to use complementary awareness creation methods other than school pupils as not all children are enrolled in school, and some may not relay the correct message to their parents. The methods of awareness creation mentioned that would help reach as many community members as possible included; local leaders who should be well informed so as to give the correct message, town criers, religious institutions and vernacular radio stations.

A 33 year old male pre-school teacher stated: “*You could do it through the provincial administration like the assistant chief, village elders. They health workers can give information. You can also use mass media, Radio, KBC- the one owned but the government. If the ministry can use radio it can reach every citizen even in the rural area.”*

Furthermore, 77% of the participants in more than three- fifths (*n* = 13) of the FGDs mentioned that use of school children for community sensitization was not effective as majority of the children did not inform the parents who only knew about the programme after the children had received the drugs. A 37 year female FGD participants from Lunga Lunga sub-county stated that:

“*For me I did not know, I was told by my children after they were treated in school*.”

In Matuga sub-county, a 41 year male FGD participant further stated:

“*I cannot say for sure that my children took the drugs, as parents we were not involved.”*

Use of school and chief’s meetings where health personnel would be invited was mentioned as the most useful method as it would provide opportunity for interaction with 80% of participants in all FGDs highlighting that they did not understand why adults were not targeted during the NSBDP and since most people did not have information about programme, some of the children received but did not swallow the drugs. A 45 year old male FGD participant in Msambweni sub-county stated that:

“*Most of the children did not take the drugs, they just went aside and threw them away*.”

A 36 year old male FGD participant in Matuga sub-county further stated that:

*“My child did not take those drugs. She ran away. There are those big kids who are mature, so they threaten the ones in nursery, class one, class two. They told them that there is a car that will come and it will be carrying doctors. So if they agreed to take the drugs, that a devil will enter them. So when the kids saw the car, they ran away.”*

### Perceptions of period of time for awareness creation

Regarding the period of time that sensitization activities for deworming were conducted 92% of the study participants indicated that the period was inadequate. Close to one-third (*n* = 13) of the opinion leaders mentioned that some areas are far to reach within the one week given and for people to understand the need for deworming.

A 40 year old male opinion leader in Matuga sub-county stated that: “*People were informed prior to the exercise that there will be ABCD, but what we didn’t know was the exact date, most people if you ask them will say they got the information from their children, they came and told us that there will be a certain exercise but they weren't informed of the date yet, so they informed the parents, the exercise was abrupt.”*

Similarly a majority of the CHEWs (*n* = 21) indicated that the one week period given for awareness creation was inadequate to cover all communities and make them understand the benefit of having their children dewormed.

Repeated awareness creation before deworming day to reach those missing the initial messages and remind those likely to forget was also mentioned as important by 80% of the participants in the FGDs. A female FGD participant stated:

“*Some children ran away and did not take the drugs because the treatment was done suddenly and the parents were not informed. It was just the children and the teachers.”*

In Matuga sub-county, a 30 year old female pre-school teacher stated that: “*I don’t think that period that I got the information was enough at all. It was not enough since it did not capture the children that are usually not enrolled in school.”*

### Perceptions on length of period between which information is given and when drugs are administered

Eighty eight percent of the participants in all study groups indicated that they learned of deworming one day before it took place or after their children had received the drugs. Slightly more than a quarter (*n* = 14) of the opinion leaders indicated that the period between awareness creation and deworming activity was too short. A 48 year old male village elder in Matuga Sub-county stated: “*I did not even have time to understand what the programme was all about because even the information given was too little. They just told us like today and tomorrow the distribution was being done. We should be given more time so that us as parents we are able to understand what is happening.”*

A 24 year old male CHEW in Msambweni Sub-county stated: “*Yes but it could have been more adequate if they could have been added more time before the exercise so that the village elders reach everyone*.”

A large majority of the participants in more than three-fifths of the FGDs (*n* = 13) indicated that the period between awareness creation and drug administration was short as the children were just informed one day before the treatment day. A 28 year old female in an FGD in Malindi sub-county stated that: “*If it is like today, the school children were told that tomorrow you will be given medicine*.”

## Discussion

The results reported in the current study show that majority of the community sensitization for school-based deworming was conducted through school pupils. The study results have also highlighted the importance of holding school or chief’s meetings where health workers are present and community members can interact and seek clarification about the programme and learn about prevention and control of worms. The stakeholders’ prefer that community sensitization be conducted using particularly the healthcare workers who are knowledgeable about the helminthic infections and would provide health education. Other studies on awareness creation conducted in the same region [14, 15] have highlighted the importance of fully involving the healthcare workers as frontline players in awareness creation for disease control and elimination programmes. Furthermore, a study on community’s perception of mass screening for malaria treatment in Western Kenya pointed out the importance of meetings citing these as useful for interaction and learning [16].

Results of the study also show that there are children who received but did not ingest the deworming drugs with parents reporting that some received the drugs and threw them away. This further shows that failure to sensitize the parents and have them support the programme is detrimental to its success. The teachers need to ensure that the children receive and ingest the drugs through directly observed treatment and this should be emphasized during training. Misconceptions about the drugs due to inadequate community sensitization as reported in a study on increasing coverage in mass drug administration for lymphatic filariasis elimination in Coastal Kenya [14] can negatively affect the success of the programme.

The results further demonstrate that religious institutions, town criers and vernacular radio stations are other preferred methods of awareness creation as these can be used to reach large populations and also serve as reminders. Mass media especially radio is used as a way of exposing high proportions of large populations to messages [17]. The stakeholders furthermore emphasized on the importance of using such methods citing their importance in reaching non-enrolled school age children. Similarly, a study on malaria control in Rwanda [18] identified such channels as important for creating awareness. Similar strategies have been reported to achieve sufficient coverage and impact of messages particularly for resistant individuals and disadvantaged or isolated groups [19].

Results of the study have also shown that the community members regard the information given for school-based deworming as inadequate and are of the perception that use of complementary methods with an aim of reaching majority of the community members especially the parents would be important. Creating awareness through health education though may not bring about behavior change is a good step towards control and elimination of worms [20]. It is important to give adequate knowledge to the parents so as to change poor perceptions and practices even at the community level and help prevent re-infection of the treated school-age children. Access to safe water, sanitation and hygiene and environmental conditions which require community awareness and participation have an impact on prevalence and intensity of re-infection after deworming [21]. Sustainability of disease control programmes through prevention of re-infection as reported in other studies requires the local stakeholders to be actively involved, empowered and be given responsibilities [22, 23].

The current study results also demonstrate that the stakeholders perceive the one week period within which the awareness creation was conducted as inadequate. The stakeholders request for an increase in the period of awareness creation saying that it would be important in reaching community members in far and remote areas and also increase their understanding of the importance of deworming. Results of the study have also indicated the need for informing the communities about the deworming in good time prior to the deworming activities. The study participants reported that the deworming activity was conducted too soon after the information was given thus denying parents sufficient time to understand the programme. The buy-in of stakeholders of a programme is reported to be of great value to its success in coverage as well as its sustainability [24]. Furthermore, integration of school-based deworming programme activities especially the health education aspect with other health system components using the existing workforce could help in increasing the period of awareness creation towards sustainability as prescribed by literature review on sustainability in health systems [25].

## Conclusion

Awareness creation is a key factor in the success of NSBDP implementation. For programme sustainability, local stakeholders need to be actively involved in programme planning and implementation. This study advocates for improvement in making deworming information accessible to all stakeholders and for ensuring that local leaders and health personnel play a major role in information dissemination. Increasing the number of times that the information is disseminated will help ensure that majority of the local stakeholders are aware and in support of the programme. Control of worms can only be achieved through integration of deworming, health education and use of safe water and sanitation facilities which requires involvement and empowerment of local stakeholders.

## Limitations of the Study

This was purely a qualitative study and the purpose was to assess the local stakeholders’ perceptions of the awareness creation strategies conducted during NSBDP. All the study participants were purposively selected. The results are not generalizable as the sample size of a qualitative study is not representative but can be used to build theory through tentative hypotheses. The results will inform the NSBDP management on considerations to make during planning and implementation of community sensitization activities.

## Abbreviations

CHEWs: Community health extension workers
FGDs: Focus group discussions
IDIs: In-depth interviews
KBC: Kenya broadcasting cooperation
KEMRI: Kenya Medical Research Institute
MDA: Mass drug administration
NSBDP: National school-based deworming programme
NTDs: Neglected tropical diseases
SAC: School-age children
STH: Soil-transmitted helminthes
STI: Science technology and innovation
WHO: World health organization
